# A POPULATION STUDY ON GENDER AND ETHNICITY DIFFERENCES IN GALLBLADDER DISEASE IN BRAZIL

**DOI:** 10.1590/0102-672020210002e1652

**Published:** 2022-06-17

**Authors:** João Henrique Fonseca do NASCIMENTO, Selton Cavalcante TOMAZ, Benjamim Messias de SOUZA-FILHO, Adriano Tito Souza VIEIRA, André Bouzas de ANDRADE, André GUSMÃO-CUNHA

**Affiliations:** 1 Universidade do Estado da Bahia, Departamento de Ciências da Vida - Salvador - Bahia - Brasil;; 2 Universidade Federal da Bahia, Faculdade de Medicina da Bahia - Salvador - Bahia - Brazil

**Keywords:** Gallbladder Diseases, Gallstones, Epidemiology, Gender and Health, Ethnic Distribution, Doenças da Vesícula Biliar, Cálculos Biliares, Epidemiologia, Gênero e Saúde, Distribuição por Etnia

## Abstract

Gallbladder diseases (GBD) are one of the most common medical conditions requiring surgical intervention, both electively and urgently. It is widely accepted that sex and ethnic characteristics mighty influence both prevalence and outcomes.

**AIM:**

This study aimed to evaluate the differences on distributions of gender and ethnicity related to the epidemiology of GBD in the Brazilian public health system.

**METHODS:**

DATASUS was used to retrieve patients’ data recorded under the *International Code of Diseases* (*ICD-10*) - code K80 from January 2008 to December 2019. The number of admissions, modality of care, number of deaths, and in-hospital mortality rate were analyzed by gender and ethnic groups.

**RESULTS:**

Between 2008 and 2019, a total of 2,899,712 patients with cholelithiasis/cholecystitis (K80) were admitted to the hospitals of the Brazilian Unified Health System, of whom only 22.7% were males. Yet, the in-hospital mortality rate was significantly higher in males (15.9 per 1,000 male patients) than females (6.3 per 1,000 female patients) (p<0.05). Moreover, men presented a significantly higher risk of death (RR=2.5; p<0.05) and longer hospital stay (4.4 days vs. 3.3 days; p<0.05) than females. Compared to females, men presented a higher risk of death across all self-declared ethnic groups: whites (RR=2.4; p<0.05), blacks (RR=2.7; p<0.05), browns (RR=2.6; p<0.05), and Brazilian Indians (RR=2.13; p<0.05).

**CONCLUSION:**

In the years 2008-2019, women presented the highest prevalence of hospital admissions for GBD in Brazil, and men were associated with worse outcomes, including all ethnic groups.

## RESUMO - RACIONAL

Doenças da vesícula biliar (DVB) são uma das condições médicas mais comuns que requerem intervenção cirúrgica, tanto eletiva como urgente. É amplamente aceito que o sexo e as características étnicas podem influenciar a prevalência e os desfechos.

**OBJETIVO:**

Avaliar as diferenças nas distribuições de gênero e etnia relacionados à epidemiologia da DVB no sistema público de saúde brasileiro.

**MÉTODOS:**

O DATASUS foi usado para elencar os dados de pacientes registrados no Código Internacional de Doenças (CID-10) sob o código K80, de janeiro de 2008 a dezembro de 2019. O número de admissões, caráter de atendimento, número de óbitos e taxa de mortalidade hospitalar foram analisados por gênero e por etnia.

**RESULTADO:**

Entre 2008 e 2019, 2.899.712 pacientes com colelitíase/colecistite (K80) foram admitidos em hospitais do Sistema Único de Saúde (SUS), dos quais apenas 22,7% eram do sexo masculino. Ainda assim, a taxa de mortalidade intra-hospitalar masculina (15,9:1.000 pacientes homens) foi significativamente maior do que a feminina (6,3:1.000 pacientes mulheres) (p<0,05). Ademais, homens apresentaram risco de morte significativamente maior em comparação às mulheres (RR=2,5; p<0,05) e maior tempo de internação hospitalar (4,4 dias *versus* 3,3 dias; p<0,05). Em comparação ao sexo feminino, homens apresentaram maior risco de morte em todos os grupos étnicos autodeclarados: brancos (RR=2,4; p<0,05), negros (RR=2,7; p<0,05), pardos (RR=2,6; p<0,05) e indígena (RR=2,13; p<0,05).

**CONCLUSÃO:**

Nos anos de 2008-2019, as mulheres apresentaram as maiores prevalências de internações hospitalares por DVB no Brasil, porém, os homens foram associados a piores desfechos, inclusive entre todos os grupos étnicos.

## INTRODUCTION

Gallbladder disease (GBD) is one of the most common gastrointestinal disorders and the leading causes of hospital admissions and surgery worldwide, responsible for over 700,000 cholecystectomies per year in the United States^4^ ^,^ ^19^ ^,^ ^25^. Global prevalence varies from 3% to 47%, with an estimated risk of 7.9% in men and 16.6% in women^4^ ^,^ ^25^ ^,^ ^29^. In Brazil, GBD affects about 10% of the general population, being accountable for over 185,000 cholecystectomies yearly^6^ ^,^ ^9^. The term GBD refers to a diverse spectrum of conditions affecting the biliary tract, which is frequently associated with the presence of gallstones (cholelithiasis)^19^ ^,^ ^29^. Ultrasound study of the gallbladder is considered the gold-standard method to diagnose GBD^19^ ^,^ ^21^.

In Western countries, the disease is mainly caused by cholesterol stone formation (>85%), and approximately 95% of cases of cholecystitis are the result of obstructive lithogenesis^4^ ^,^ ^13^. Pathogenesis of gallstone disease is considered multifactorial and results from a complex interaction between genetic, hormonal, physiological, behavioral, and environmental factors^10^ ^,^ ^27^ ^,^ ^29^. Several authors have demonstrated the relationship between GBD and metabolic disorders such as obesity, hypertriglyceridemia and type 2 diabetes, family history, physical inactivity, tobacco and alcohol consumptions, age (directly proportional), gender (female > male), animal fat-rich diets, rapid weight loss, and drugs^10^ ^,^ ^19^ ^,^ ^24^ ^,^ ^29^. About one in five stones carriers will develop clinical manifestations, and the potential risk for the progression to symptomatic disease is briefly explained by the “4F” rule: Fatty, Forty, Fertile, and Female^10^ ^,^ ^24^ ^,^ ^25^.

There is a strong and well-established direct association between body mass index (BMI) and the incidence of the disease, in which a high BMI poses an up to 7-fold increased risk for cholelithiasis^21^. Age is also a major risk for GBD, achieving relatively high rates in individuals over 40 years of age, possibly as a result of cumulative stone formation^21^. Moreover, ethnic differences seem to influence the variation of prevalence, which previous studies have suggested that the greatest risks occur among Amerindians and that the black skin population presents the lowest prevalence of GBD^11^ ^,^ ^13^. Nevertheless, associative studies between ethnicity and gallstone diseases in Brazil are scarce.

Therefore, given the prevalence of GBD, the scarcity of reports in the literature comparing ethnicity and sex-related outcomes and, since epidemiological data are indispensable for diagnostic, therapeutic, and public policies strategies, this study aimed to assess the ethnic distribution and gender disparities in the GBD among patients attending public hospitals in the Brazilian Unified Health System (Sistema Único de Saúde - SUS).

## METHODS

This population-based, retrospective, and longitudinal study, carried out with secondary data from a government database, evaluated the ethnic influences and gender discrepancies as specific risk factors in hospital morbidity and mortality for GBD in Brazil. The Unified Health System Department of Informatics (*Departamento de Informática do Sistema Único de Saúde* - DATASUS) is a public online data platform, managed by the Ministry of Health, along with the state and municipal health secretariats (available for online access at http://datasus.saude.gov.br/). Data on morbidity were collected through the Hospital Information Systems (*Sistema de Informação Hospitalares em Morbidade Hospitalar* - SIH) from DATASUS, which gathers most of the information regarding the numbers of hospitalizations and hospital admission authorization forms (*Autorização de Internação Hospitalar* - AIH), length of stay, list of diseases, and patient outcomes. DATASUS platform defines *hospital admission* for an inpatient as remaining in hospital for more than 24 h; thus, day-hospital approaches were not included in the analyses. All data were stratified geographically by the patient place of residence.

Data were collected based on the *International Disease Classification* (*10th Revision - ICD-10*), using the K80 code, recorded on DATASUS as “Cholelithiasis and Cholecystitis,” from January 2008 to December 2019. To perform this investigation, we analyzed the following variables: gender, self-reported ethnicity (i.e., white, black, brown, and Brazilian Indians), cases above and below the age of 40, total number of hospital admissions and number of admissions by gender and by ethnicity, emergency and elective interventions, total number of in-hospital deaths and the in-hospital mortality rate, and average length of stay.

Normality of the variables was assessed by the Shapiro-Wilk test and the Q-plot. Homogeneity of the compared groups’ variances was assessed using the Levene’s Test for Equality of Variances. Descriptive statistics such as mean, standard deviation (SD), median, interquartile range (IQR: Q1-Q3), relative risk (RR), and confidence intervals (CI) were used to describe numbers and proportions of admissions, gender- and ethnic-related risk of deaths, and length of in-hospital stay.

Fisher’s exact test and chi-square test with Yates’ continuity correction were used to compare proportions between two groups. Depending on the normal or non-normal distribution of the variables, a Mann-Whitney U test and a Student’s t-test for independent samples were also used when appropriate to compare differences between groups. To assess the data variance along time, percentage variation was calculated between the years applying the following formula: [(next year − previous year)/previous year] ×100, in order to identify the stability, increase, or decrease of the numbers. Adjusted r² values were obtained using linear regression to evaluate the variance of trends, considering a p<0.05 result as significant.

Data management and the statistical analysis were conducted using the Microsoft Office Excel 2019 software (Microsoft, Redmond, WA, USA), the BioEstat software (*Instituto de Desenvolvimento Sustentável Mamirauá*, version 5.3), and the R software (RStudio, Inc. - R Foundation for Statistical Computing, version 4.0.3), a free from charge software for data analysis.

Approval of the Ethics Committee in Research is considered dispensable, since secondary data were obtained from the public domain and online database, without individual identification of patients.

## RESULTS

From 2008 to 2019, a total of 2,899,712 patients with cholelithiasis/cholecystitis were admitted to the hospitals of the Brazilian Unified Health System, of whom 657,586 (22.7%) were males and 2,242,126 (77.3%) were females (Table 1). Women presented an average of 186,844 (SD=20,642.6) annual hospital admissions, whereas men counted an average of 54,799 (SD=7,198.5) hospitalizations per year. Female-to-male proportion was 3.4:1 (p<0.05). Table 1 presents the total number of hospital admissions per year by gender, in which a trend toward increase is seen for overall cases (r²=0.958), also observed in both men (r²=0.947) and women (r²=0.953), with average growth of +4.4% (SD=0.031) and +3.6% (SD=0.026) per year, respectively. Average length of stay for GBD was 3.6 days per patient and showed a trend toward shortening from 2008 to 2019 (r²=0.9798), with an average shortening of −1.3% (SD=0.01) per year. Men presented a significantly longer length of stay compared to women (4.4 days vs. 3.3 days; p<0.05).

**Table 1 -:** Number of hospital admissions and in-hospital deaths due to (K80) cholelithiasis and cholecystitis in the Unified Health System, by gender (2008-2019)

	Hospital admissions			In-hospital death		
	Overall	Male	Female	Overall	Male	Female
2008	193,997	42,847	151,150	1,787	763	1,024
2009	209,151	47,965	61,186	2,049	864	1,185
2010	222,994	50,140	172,854	2,107	941	1,166
2011	225,690	50,251	175,439	2,120	876	1,244
2012	231,003	51,692	179,311	2,032	861	1,171
2013	236,461	52,278	184,183	2,076	850	1,226
2014	249,403	55,196	194,207	1,983	830	1,153
2015	247,860	56,103	191,757	1,993	838	1,155
2016	251,758	57,546	194,212	2,144	905	1,239
2017	261,115	60,599	200,516	1,986	846	1,140
2018	280,124	64,766	215,358	2,132	926	1,206
2019	290,156	68,203	221,953	2,146	947	1,199
**Total**	**2,899,712**	**657,586**	**2,242,126**	**24,555**	**10,447**	**14,108**
**Mean**	**241,643**	**54,799**	**186,844**	**2,046**	**871**	**1,176**
*SD*	27,769.93	7,198.52	20,642.56	102.14	52.68	58.85
**Median**	**242,161**	**53,737**	**187,970**	**2,063**	**863**	**1,178**
*IQR (Q1-Q3)*	225,016.0-254,097.3	50,223.3-58,309.3	174,792.8-195,788.0	1,991.3-2,123.0	844.0-910.3	1,154.5-1,211.0
***r²***	***0.9582***	***0.9477***	***0.9534***	***NS***	***NS***	***NS***

NS: not significant.

There were 24,555 deaths from 2008 through 2019, with 14,108 (57.4%) fatalities counted among women, with an average of 1,176 (SD=58.8) deaths per year, whereas 10,447 (42.6%) fatalities were related to men, with an average of 871 (SD=52.7) deaths per year. Female-to-male death proportion was 1.35:1 (p<0.05). Overall in-hospital mortality rate was 8.5 deaths per 1,000 patients, and the in-hospital mortality rate of male (15.9 obits per 1,000 patients) was significantly higher than the rate of females (6.3 obits per 1,000 female patients) (p<0.05). Moreover, males presented a significantly higher risk of death compared to females (RR=2.5, 95%CI 2.4-2.6; p<0.05).

As for the modalities of care (Table 2), 43.9% (median=103,854, IQR: 101,112-112,281) of patients were managed on an emergency basis, while 56.1% (median=137,226, IQR: 121,397-148,324) were treated electively (Mann-Whitney U=16.00; p<0.05). In the elective modality of care, there were 1,627,151 hospitalizations (81.75% females and 18.25% males), with a mean of 135,596 (SD=23,443.8) elective admissions per year. In these cases, female patients had a mean of 110,863 (SD=18,360.83) annual elective admissions and added up 1,711 (60.4%) deaths in the elective group, whereas the annual mean of elective admissions of male patients was 24,733 (SD=5,128.04) hospitalizations per year and a total of 1,124 (39.6%) deaths (Table 2). Male in-hospital mortality rate in elective treatment (4.0 deaths per 1,000 elective male admission) was significantly higher than the rate of females (1.3 deaths per 1,000 elective female admission) (p<0.05).

**Table 2 -:** Management modalities of (K80) cholelithiasis and cholecystitis in the Unified Health System, by gender (2008-2019)

	Elective				Emergency			
	Hospital admitions		In-hospital death		Hospital admitions		In-hospital death	
	*Male*	*Female*	*Male*	*Female*	*Male*	*Female*	*Male*	*Female*
2008	16,040	77,794	91	142	26,734	73,173	671	882
2009	19,631	88,852	89	148	28,334	72,334	775	1,037
2010	20,412	96,628	110	151	29,728	76,226	831	1,015
2011	21,669	101,181	117	162	28,582	74,258	759	1,082
2012	22,409	104,101	85	165	29,283	75,210	776	1,006
2013	23,987	112,010	85	164	28,291	72,173	765	1,062
2014	26,303	121,840	115	146	28,893	72,367	715	1,007
2015	26,640	118,006	86	119	29,463	73,751	752	1,036
2016	25,727	112,728	105	121	31,819	81,484	800	1,118
2017	28,200	120,670	78	125	32,399	79,846	768	1,015
2018	32,039	135,693	93	115	32,727	79,665	833	1,091
2019	33,742	140,849	70	153	34,473	81,120	878	1,047
**Total**	**296,799**	**1,330,352**	**1,124**	**1,711**	**360,726**	**911,607**	**9,323**	**12,398**
**Mean**	**24,733**	**110,863**	**94**	**143**	**30,061**	**75,967**	**777**	**1,033**
*SD*	5,128.04	18,360.83	14.89	18.22	2,275.16	3,599.79	54.81	59.47
**Median**	**24,857**	**112,369**	**90**	**147**	**29,373**	**74,734**	**772**	**1,037**
*IQR (Q1-Q3)*	21,354.8-27,030.0	100,042.8-120,962.5	85.0-106.3	124.0-155.3	28,520.0-31,964.0	72,971.5-79,710.3	757.3-807.8	1,013.0-1,067.0

In the group of patients admitted on an emergency basis, the total number of hospitalizations was 1,272,333 cases (71.64% females and 28.36% males). The mean of emergency female patients was 75,967 (SD=3,599.79) hospital admissions per year, with 12,398 (57.1%) deaths. The mean of hospital admissions for emergency male patients was 30,061 (SD=2,275.16) per year, with 9,323 (42.9%) deaths. Male in-hospital mortality rate in emergency care (25.8 deaths per 1,000) was significantly higher than the rate of females (13.6 deaths per 1,000) (p<0.05). Men also presented a significantly higher risk of death in both emergency (RR=1.9, 95%CI 1.8-1.95; p<0.05) and elective (RR=2.9, 95%CI 2.7-3.17; p<0.05) modalities of care.

In the years 2008-2019, patients over 40 years of age had 1,877,262 (64.7%) hospital admissions, with a mean of 288,809.5 (SD=17,262.03) annual hospitalizations, significantly higher than patients under 40 years of age (total=1,022,477 hospital admissions; 35.3%; mean=157,304.2/year; SD=10,586.15) (p<0.05). Average annual hospitalizations of women under 40 years of age (132,487.5/year; SD=8,938.20) and over 40 years of age (212,457.2/year; SD=11,783.55) were significantly higher than those observed among men (24,816.6/year, SD=1,700.34 and 76,352.3/year, SD=5,513.60, respectively) (p<0.05 and p<0.05, respectively). Overall female-to-male hospitalization proportion was 5.3:1 for patients aged under 40 years and 2.8:1 for patients aged over 40 years (Table 3). Ages over 40 years were also associated with a longer in-hospital stay (7.1 days vs. 4.5 days; p<0.05). Men presented a significantly longer average length of stay compared to women for cases under 40 years of age (6.1 days vs. 3.6 days; p<0.05) and over 40 years of age (7.8 days vs. 6.4 days; p<0.05).

**Table 3 -:** Hospitalizations and in-hospital deaths due to (K80) cholelithiasis and cholecystitis in the Unified Health System, by gender and age (2008-2019)

	Male				Female			
	Hospital admitions		In-hospital death		Hospital admitions		In-hospital death	
	*Under 40 years*	*Over 40 years*	*Under 40 years*	*Over 40 years*	*Under 40 years*	*Over 40 years*	*Under 40 years*	*Over 40 years*
2008	10,671	32,176	70	693	56,468	94,682	67	957
2009	12,025	35,940	76	788	60,249	100,937	66	1,119
2010	12,397	37,743	79	862	64,865	107,989	68	1,098
2011	12,256	37,995	50	826	66,082	109,357	61	1,183
2012	12,564	39,128	62	799	68,214	111,097	65	1,106
2013	12,696	39,582	56	794	70,878	113,305	73	1,153
2014	13,426	41,770	46	784	75,588	118,619	66	1,087
2015	13,704	42,399	55	783	74,733	117,024	68	1,087
2016	14,061	43,485	43	862	76,653	117,559	65	1,174
2017	15,295	45,304	53	793	78,357	122,159	75	1,065
2018	15,786	48,980	53	873	83,629	131,728	71	1,135
2019	16,427	51,788	53	895	85,453	136,516	73	1,127
**Total**	**161,308**	**496,290**	**696**	**9,752**	**861,169**	**1,380,972**	**818**	**13,291**
**Mean**	**13,442.3**	**41,357.5**	**58.0**	**812.7**	**71,764.1**	**115,081.0**	**68.2**	**1,107.6**
*SD*	1,700.3	5,513.6	11.5	54.8	8,938.2	11,783.5	4.1	59.4
**Median**	**13,061.0**	**40,676.0**	**54.0**	**796.5**	**72,805.5**	**115,164.5**	**67.5**	**1,112.5**
*IQR (Q1-Q3)*	12,361.8-14,369.5	37,932.0-43,939.8	52.3-64.0	787.0-862.0	65,777.8-77,079.0	109,015.0-119,504.0	65.8-71.5	1,087.0-1,139.5

Data regarding deaths (n=24,557) showed that most of the fatalities was counted in patients above the age of 40 (93.8%), with a mean of 3,545.1 (SD=106.13) deaths per year, significantly higher than the annual mean observed in patients under the age of 40 (mean=232.9; SD=12.02) (p<0.05). Age over 40 and hospitalization for GBD predicted an overall higher risk of death (RR=8.3, 95%CI 7.8-8.7; p<0.05), and the same result was also observed for both males (RR=4.5, 95%CI 4.2-4.9; p<0.05) and females (RR=10.1, 95%CI 9.4-10.8; p<0.05), compared to males and females under the age of 40 (Table 4). Women were accountable for the highest annual means of deaths, both for cases aged under 40 years (female=125.8 deaths/year, SD=4.09 vs. male=107.1 deaths/year, SD=11.47) and over 40 years (female=2,044.8 deaths/year, SD=59.43 vs. male=1,500.3 deaths/year, SD=54.79). Despite the high number of deaths associated with women, men exhibited a higher risk of death for both cases under (RR=4.54, 95%CI 4.1-5.0; p<0.05) and over (RR=2.0, 95%CI 1.98-2.1; p<0.05) the age of 40, compared to females.

**Table 4 -:** Comparative analysis between gender and age for relative risk (RR) of death due to (K80) cholelithiasis and cholecystitis in the Unified Health System (2008-2019)

	RR	95%CI	p-value
Male			
Over 40 years of age	4.5	4.2-4.9	<0.05
Under 40 years of age	1.0*	-	
Female			
Over 40 years of age	10.1	9.4-10.8	<0.05
Under 40 years of age	1.0*	-	
Gender under 40 years of age			
Male	4.54	4.1-5.0	<0.05
Female	1.0*	-	
Gender over 40 years of age			
Male	2.0	1.98-2.1	<0.05
Female	1.0*	-	

As for ethnicity, most hospital admissions were from self-declared white patients (total=1,063,538, mean=88,628 hospitalizations/year; SD=8,947.70), followed by self-declared brown patients (total=901,037, mean=75,086 hospitalizations/year; SD=20,790.20), self-declared black patients (total=83,686, mean=6,974 hospitalizations/year; SD=1,624.03), and self-declared Brazilian Indians patients (total=4,957, mean=413 hospitalizations/year; SD=159.25). Similar results were observed concerning deaths, in which self-declared white patients were accountable for 9,530 deaths (mean=794/year; SD=29.39), followed by self-declared browns (n=6,547; mean=546/year; SD=136.94), self-declared blacks (n=809; mean=67/year; SD=13.30), and self-declared Brazilian Indians (n=31; mean=3/year; SD=2.15). Self-declared white patients presented the highest overall risk of death compared to non-self-declared white patients (RR=1.2, 95%CI 1.16-1.22; p<0.05), followed by self-declared blacks compared to non-self-declared black patients (RR=1.18, 95%CI 1.1-1.2; p<0.05). Self-declared brown ethnicity was identified as a protective factor (RR=0.81, 95%CI 0.78-0.83; p<0.05). No association was observed among the self-declared Brazilian Indians (p=0.12).

The ethnicity data and proportions broken down by gender are shown in Table 5. Self-declared black men and black women presented the highest risk of death compared to non-self-declared black respective gender (RR=1.2, 95%CI 1.1 - 1.4, p<0.05 and RR=1.16, 95%CI 1.0-1.2, p<0.05, respectively). As previously observed, self-declared brown ethnicity was also identified as a protective factor among brown men (RR=0.92, 95%CI 0.8-0.96; p<0.05) and brown women (RR=0.8, 95%CI 0.7-0.8; p<0.05). No association was observed among the self-declared Brazilian Indians for both males (p=0.18) and females (p=0.37). Table 6 shows that males presented a significantly higher risk of deaths in all ethnic groups.

**Table 5 -:** Number of hospital admissions and in-hospital deaths due to (K80) cholelithiasis and cholecystitis in the Unified Health System, by gender and ethnicity (2008-2019)

	Hospital admitions								In-hospital death							
	*White*		*Black*		*Brown*		*Indians*		*White*		*Black*		*Brown*		*Indians*	
	M	F	M	F	M	F	M	F	M	F	M	F	M	F	M	F
2008	18,323	59,231	1,100	4,173	8,705	34,436	191	529	330	441	19	32	124	154	4	3
2009	18,986	59,341	1,245	4,529	10,745	39,915	114	382	357	471	37	38	171	271	2	3
2010	19,169	61,758	1,283	4,797	12,212	45,936	96	349	329	474	31	32	218	235	1	0
2011	19,787	62,665	1,229	4,703	12,556	48,697	62	183	365	449	24	37	181	298	1	0
2012	19,403	62,029	1,271	4,448	12,960	49,429	34	189	311	418	26	30	185	261	0	0
2013	20,419	65,044	1,249	4,886	14,505	56,740	71	266	336	473	21	28	203	309	0	4
2014	22,107	70,581	1,386	5,285	16,384	63,263	70	227	361	459	23	37	234	328	0	2
2015	22,100	68,807	1,367	5,131	17,415	64,826	73	214	332	434	42	37	248	343	0	1
2016	22,647	70,205	1,597	5,691	18,480	67,909	79	219	369	432	20	45	267	414	2	3
2017	23,519	71,770	1,838	6,684	20,405	73,584	94	343	344	430	26	48	270	366	1	1
2018	25,035	76,090	2,011	7,332	22,258	80,642	107	436	366	424	43	46	313	406	0	2
2019	26,374	78,148	2,287	8,164	23,953	85,082	142	487	378	447	33	54	323	425	1	0
**Total**	**257,869**	**805,669**	**17,863**	**65,823**	**190,578**	**710,459**	**1,133**	**3,824**	**4,178**	**5,352**	**345**	**464**	**2,737**	**3,810**	**12**	**19**
**Mean**	**21,489.1**	**67,139**	**1,488.58**	**5,485**	**15,881.5**	**59,205**	**94.42**	**319**	**348**	**446**	**29**	**39**	**228**	**318**	**1**	**2**
*SD*	2,571.14	6,393.27	368.57	1,257.27	4,752.68	16,049.6	41.21	120.28	20.61	19.55	8.38	7.99	59.41	80.43	1.21	1.44
**Median**	**21,259.5**	**66,926**	**1,325**	**5,009**	**15,444.5**	**60,002**	**86.5**	**305**	**351**	**444**	**26**	**37**	**226**	**319**	**1**	**2**
*IQR (Q1)*	19,344.5	61,961.3	1,248.0	4,659.5	12,470.0	48,006.8	70.7	217.8	331.5	431.5	22.5	32.0	184.0	268.5	0	0
*IQR (Q3)*	22,865.0	70,878.3	1,657.2	5,939.3	18,961.2	69,327.8	108.7	395.5	365.3	462.0	34.0	45.3	267.8	376.0	1.3	3.0

**Table 6 -:** Comparative analysis between gender and ethnicity for relative risk (RR) of death due to (K80) cholelithiasis and cholecystitis in the Unified Health System (2008-2019)

	RR	95%CI	p-value
White			
Male	2.4	2.3-2.5	<0.05
Female	1.0*	-	
Black			
Male	2.7	2.3-3.1	<0.05
Female	1.0*	-	
Brown			
Male	2.6	2.5-2.8	<0.05
Female	1.0*	-	
Brazilian indians			
Male	2.13	1.03-4.4	<0.05
Female	1.0*	-	

## DISCUSSION

Cholelithiasis is the presence of one or more stones in the gallbladder and its complications, such as cholecystitis, pancreatitis, cholangitis, and even gallbladder cancer, represent an important public health problem worldwide^16^ ^,^ ^19^. Despite this, only a few Brazilian population-based studies on the prevalence and epidemiological implications of GBDs have been published. GBD constitutes a major concern for the Unified Health System and accounts for over 240,000 hospital admissions every year in Brazil. This study aimed to evaluate gender and ethnic influences associated with cholelithiasis/cholecystitis in order to obtain knowledge on patient outcomes amidst the Brazilian population.

As a result of our investigation, we identified that female gender, self-declared white ethnicity, age over 40, and elective treatment are important risk factors in the overall in-hospital prevalence of GBDs nationwide. Brazilian women were accountable for over 2 million (77.3%) hospital admissions in the years 2008-2019, with more than 150,000 hospitalized women every year and a total of 57.1% (12,398) of all deaths. Similar results were reported in a large cross-sectional 65,511-patient study performed in the United States in 2010, which showed a higher prevalence of gallstone disease in the female group (70.7%; p<0.0001)^15^. Sevinç et al. also reported a female/male proportion of 1.7:1 in a retrospective study with 330 patients presenting with gallstones to the General Surgery Department at a University Hospital in Turkey^24^. Furthermore, the ELSA-Brazil study (Brazilian Longitudinal Study of Adult Health - *Estudo Longitudinal de Saúde do Adulto*) evaluated the sociodemographic and clinical characteristics associated with cholecystectomy on the follow-up of 5,061 participants in São Paulo and demonstrated that the female gender was an independent risk factor (OR=2.83), even with adjustment for age (OR=2.85)^1^.

Several authors have concluded that women have higher risk of gallstone formation than do men^10^ ^,^ ^13^ ^,^ ^21^ ^,^ ^24^ ^,^ ^25^ ^,^ ^26^. The observed gender-related discrepancies in GBD prevalence may be due to the effects of endogenous estrogen, which, in turn, may reflect on the secretion and biosynthesis of hepatic cholesterol^25^. Previous studies have suggested that the interaction of 17β-estradiol with the nuclear estrogen receptor (ESR1) leads to a cholesterol hypersecretion into bile^21^ ^,^ ^28^ ^,^ ^29^. Estrogen also enhances the activity of hydroxyl-3-methylglutaryl coenzyme A (HMG-CoA) reductase, which induces biliary cholesterol hypersecretion^21^ ^,^ ^25^. Based on this, the estrogen increases biliary cholesterol secretion leading to cholesterol hypersaturation of bile, which favors lithogenesis^25^ ^,^ ^29^. Exogenous estrogen also seems to contribute to gallstone formation, since hormone replacement therapy and oral contraceptives containing estrogen are associated with a high incidence of GBD^19^. Although gallstones are rare in children, the female gender appears to be a risk characteristic even for pediatric symptomatic GBD, as seen in a retrospective cross-sectional study in the United States, with 404 patients aged between 0.6 and 18 years, in which 73% were girls^17^. Besides sex hormones, the number of gestations also impacts the incidence of the disease. Pregnancy causes a decrease in the gallbladder motility during the third trimester favoring the formation of stones and, since there is a cumulative effect, the risk is increased in multiparous females^19^ ^,^ ^21^.

Although women represented the largest number of cases in our study, men presented higher chances of worse outcomes. Some studies help to understand these observations based on the biochemical composition of gallstones and differences between sexes. The composition of stones might influence patient prognosis since cholesterol gallstones have less chance of bacterial colonization and, hence, less risk of progressing to sepsis^5^ ^,^ ^8^ ^,^ ^14^ ^,^ ^30^. A study^8^ from Italy evaluated the composition of the stones from 960 patients who underwent surgery for GBD and reported that only 13.5% of patients with cholesterol stones had positive blood cultures, worth mentioning that most non-cholesterol stones have been demonstrated to harbor bacteria^14^ ^,^ ^30^. Women, especially between the menarche and menopause, are more likely to develop gallstones and tend to have a higher percentage of cholesterol in their composition, when compared to men. In a New Zealand study by Stringer, the analysis of specimens from 107 patients revealed that 60% of stones from female carriers had more than 70% of cholesterol in their composition, whereas the mean fraction of cholesterol in the composition of the stones from male patients was only 37.5%^5^, which might represent a protective factor to women.

Our results associated men to a higher overall in-hospital mortality rate (15.88 vs. 6.29 by 1,000 patients; p<0.05) and a higher risk of death by GBD (RR=2.5; p<0.05) compared to women, and similar proportions were also observed among patients below the age of 40 (4.3 vs. 0.9 per 1,000 patients; RR=4.5; p<0.05) and above the age of 40 (19.6 vs. 9.6 per 1,000 patients; RR=2.0; p<0.05). Despite the clearly increased prevalence of GBD in females, the literature shows that males present worse outcomes and seem more likely to be correlated with higher rates of conversion from laparoscopy to an open procedure, longer hospital stay, higher risk of complications in laparoscopic cholecystectomy, and higher risk of death^9^ ^,^ ^27^. A study in Greece showed that, compared to women, men presented longer operative time (45 min vs. 40 min; p<0.05), more conversion from laparoscopic to open cholecystectomy (8.7% vs. 4.6%; p<0.05), higher postoperative complications rate (3.7% vs. 1.6%; p<0.05), and also more frequent presence of empyema at clinical presentation (7.4% vs. 4.0%; p<0.05)^5^. A 5-year retrospective study from Germany showed that male cases presented more frequently both gangrenous (29.0% vs. 20.3%; p<0.05) and necrotizing cholecystitis (33.3% vs. 10.1%; p<0.05) and a higher median of intraoperative blood loss (250 mL vs. 180 mL; p<0.05) compared to female cases^3^. In Brazil, Felício et al. demonstrated that the number of deaths was higher among men in both elective (0.0006% vs. 0.0004%) and urgent (0.0027% vs. 0.0022%) cholecystectomies in a comparative study on acute cholecystitis^12^.

The present investigation revealed a remarkable higher risk of death associated with the age over 40 (RR=8.3; p<0.05). Regardless of age, males experienced higher mortality risks, for both men below and above the age of 40 (RR=4.54 and RR=2.0, respectively), compared to women. Further, in the analyses of age groups within the same gender, males and females over 40 years exhibited notably higher risks of deaths compared to the cases under the age of 40 (RR=4.5 and RR=10.1, respectively). Several publications reported that the incidence of stones and GBD increases directly proportional to age, escalating after ages of 40 to become 4-10 times more likely to occur^19^ ^,^ ^21^ ^,^ ^28^. The literature shows reduced bile acid biosynthesis as an underlying cause for increased cholesterol saturation in the elderly, which ultimately contribute to gallstone formation and highest risks among people above the fifth decade of life^19^. Sandblom et al. reported similar observations in a Swedish study, which identified a mortality rate two times higher among people between 50 and 70 years of age when compared to the general population and up to seven times higher when compared with individuals above the age of 70^23^. In 2015, Nimptsch et al. also identified age as an independently risk factor, with a mortality rate 10 times higher for individuals over 65 years old, in a German study with 731,000 cholecystectomies in the years 2009-2013^20^.

Furthermore, our analysis revealed that Brazilian male patients had longer hospitalization time (4.4 days vs. 3.3 days; p<0.05), including in the comparison between male and female cases under 40 years of age (6.1 days vs. 3.6 days; p<0.05) and over 40 years of age (7.8 days vs. 6.4 days; p<0.05). Similar findings have been previously reported by Ambe et al. in a study with 779 patients (462 females and 317 males) undergoing laparoscopic cholecystectomy for acute cholecystitis in Germany, evidencing that hospital stay was significantly longer among male patients (6 days, SD=1-54 days vs. 5 days, SD=2-42 days)^2^. Although small, a similar difference was also observed by Botaitis et al, when analyzing 2,000 patients (1,515 females and 485 males) undergoing cholecystectomy for GBDs, of whom men presented a longer postoperative stay (3.4 days, SD=1.1 vs. 3.2 days, SD=0.9; p<0.05)^5^. A longitudinal study placed in the Department of General Surgery at a University Hospital in Poland evaluated 504 patients (178 males and 326 females) admitted and scheduled to cholecystectomy due to GBDs and showed that male cases had a higher mean of length of hospital stay (92.5 h vs. 80.7 h)^22^. In Brazil, a retrospective study evaluated 250,439 patients undergoing cholecystectomies from 2009 to 2014 and demonstrated that men had longer in-hospital stay in days (5.3 days vs. 4.4 days)^12^.

These findings have been confronted by other authors. A retrospective study in Germany with 138 patients presenting with acute cholecystitis (69 males and 69 females) demonstrated no differences in the median length of postoperative in-hospital stay between males and females (6 days in both groups; p=0.27)^3^. In 2019, a 1,645-laparoscopic cholecystectomies patients investigation (540 males and 1,105 females) placed on the state of Paraná (Brazil) also reported no difference in the length of hospital stay between sexes (1.14 days vs. 1.07 days; p=0.206)^9^.

Ethnic background is a well-known non-modifiable risk factor for gallstone diseases^28^. It is important to emphasize that there is no previous nationwide study that statistically compared the prevalence of GBD and relative risks across ethnic groups in Brazil until the present moment. Our data showed that the white group was the most prevalent in the general population (36.6%), with a mean of 88,628 (SD=8,947.7) hospital admissions per year, also observed in both white males (39.2%; mean=21,489, SD=2,571.1 hospitalizations/year) and white females (35.9%; mean=67,139, SD=6,393.3 hospitalizations/year). A study with 14,238 individuals in the United States, in association with the Third National Health and Nutrition Examination Survey, evidenced that the prevalence of GBD was significantly higher among white non-Hispanic men (8.6%; p<0.001) and women (16.6%; p<0.001) compared to the black non-Hispanic gender groups (5.3% and 13.9%, respectively), and observed a significantly reduced risk of GBD for non-Hispanic blacks men and women (OR=0.58 and OR=0.66, respectively; p<0.001) compared to the whites^11^. Ingraham et al performed an investigation on the risk factors associated with 65,511 cholecystectomies recorded at the American College of Surgeons National Surgical Quality Improvement Program Hospitals database and reported a significantly higher prevalence of GBD among the white group compared to the black group of patients (65.5% vs. 9.4%; p<0.0001)^15^.

Everhart et al., by reviewing the possible reasons for low GBD prevalence found among black people, discussed that U.S. blacks present a lower gallbladder cholesterol saturation index than whites and that African-born blacks may have better gallbladder motor function than European whites^11^. Despite the high prevalence of GBD among the Brazilian self-reported white skin group, our results revealed that the black population presented the highest average mortality rate of 9.8 deaths by 1,000 patients (p<0.05), also higher among black males (19.3 deaths per 1,000 patients) and black females (6.6 deaths per 1,000 patients), compared to the non-black respective gender groups. In addition, Brazilian blacks presented a higher risk of death compared to non-black individuals, both among men (RR=1.2; p<0.05) and women (RR=1.16; p<0.05). These findings cast a new (or recurring) light on social issues and their impacts on the prevalence, risks of GBD-related death, and ethnicity in the country. Our results might suggest bias on the actual prevalence and risk regarding this particular ethnic group since black people experience less access to medical care due to economic disparities, similar to some other parts of the world^11^, and this might make this population vulnerable to worse outcomes in Brazil.

While the prevalence of GBD is high among Canadian Indians and American Indians, up to 62% and 73%, respectively^28^, the Brazilian indigenous group accounted for only 0.17% of total hospital admissions. However, Alves et al showed that indigenous ethnicity was a risk factor for the need to undergo cholecystectomy during the first 2 years of follow-up in a cohort study with 4,716 participants in São Paulo (Brazil)^1^. This low percentage of Brazilian Indians has been challenged by several authors who observed a high prevalence of gallstones in other aboriginal populations of South America. A report from Chile found an important prevalence of GBD among Chilean Mapuche Indians (35.2%), higher than in the residents of urban Santiago (27.5%), and also a high prevalence in the Maoris of Easter Island (20.9%)^11^ ^,^ ^18^. A genome-wide association study with samples from admixed Mapuche Native Americans from Chile revealed that variants within the *ABCG8* and *TRAF3* genes confer risk to gallstone disease and gallbladder cancer in the admixed Indian population^7^. The *ABCG8* polymorphism is associated with a hepatic Sterolin transporter, which is linked to a hypersecretion of cholesterol-saturated bile, and the *TRAF3* is a member of the tumor necrosis factor receptor protein family that mediates inflammatory responses correlated to cholecystitis - a known hallmark of GBD^7^. In contrast to these observations, it is important to consider that the low prevalence of GBD among the Brazilian Indians of our sample might be a result of inadequate access to public health services of secondary and tertiary care.

As already stated, research comparing the risks of gallstone disease and ethnic groups have rarely been performed^11^, but our results demonstrated that male cases were at greater risk of death among whites (RR=2.4; p<0.05), blacks (RR=2.7; p<0.05), browns (RR=2.6; p<0.05), and Brazilian Indians (RR=2.14; p<0.05), compared to females. These positive correlations reinforce the suggestion of a higher risk of death associated with men, even under ethnic comparation. Numerous authors have evaluated the correlations of ethnicity and gender on the epidemiology of gallstones^2^ ^,^ ^3^ ^,^ ^5^ ^,^ ^7^ ^,^ ^10^ ^,^ ^11^ ^,^ ^13^ ^,^ ^18^ ^,^ ^21^ ^,^ ^22^ ^,^ ^27^ ^,^ ^28^, but there is an important lack of studies that focus on cross-analyzing the risks of death and the influence of sex and ethnic trait on GBDs.

Several limitations exist within the scope of our study. One of the most relevant limitation is the non-differentiation of the disorders regarded the *ICD-10* K80 code, which could be separately classified as cholelithiasis with acute cholecystitis (with or without obstruction) or with chronic cholecystitis, cholelithiasis without cholecystitis, calculus of bile duct with cholangitis (with and without obstruction), and calculus of gallbladder and bile duct with cholecystitis and without cholecystitis, among others. Besides, the database did not provide information on postoperative complications. Understanding the correlations of specific gallbladder disorders, post-treatment complications, and the data on patient gender/ethnicity could evidence pivotal roles of these implications in Brazilian epidemiology.

DATASUS does not provide critical information on the profile of cases, such as body mass index, nutritional status, daily dietary composition, history and number of pregnancies, past surgeries (e.g., bariatric surgery), and previous postoperative complications. Further, the relationship between cholelithiasis/cholecystitis and lifestyle conditions (i.e., smoking status and alcohol consumption, physical activities routines, comorbidities, and drug use) or stones analysis (i.e., number and size of gallstones, biochemical composition, and bacterial colonization study) were not assessed in our research as the information could not be retrieved from the database. Another relevant limitation is the difficulty of establishing the real genetic/ethnic-related contribution to GBD in Brazil due to the intense ethnic admixture of the population and the “self-declared” character of these collected data, in which the phenotype may not represent the actual genetic load influence on the disease from each patient.

## CONCLUSION

Women presented the highest prevalence of hospital admissions for GBDs in Brazil from 2008 to 2019. Yet, male patients were correlated with a longer length of stay, a higher risk of death, and a higher in-hospital mortality rate, regardless of being under or over 40 years of age. However, cases above the age of 40 presented a higher risk of death, independent of gender. Self-declared white population showed the highest prevalence of GBD in the years 2008-2019, but self-declared black ethnicity was associated with higher risks of death and higher in-hospital mortality rates. Self-declared brown ethnicity was identified as a protective factor for the fatality of GBD. Being male was also associated with a higher risk of death in all ethnic groups.
